# Anti-biofilm activities and antibiotic synergy of naturally occurring compounds against drug-resistant rapidly growing mycobacteria

**DOI:** 10.1128/spectrum.00199-24

**Published:** 2024-06-27

**Authors:** Ya-Ling Huang, Chen-Hsiu Huang, Yu-Chieh Huang, Chun-Lun Yen, Chun-Ru Hsu

**Affiliations:** 1 Department of Laboratory Medicine, E-Da Hospital, I-Shou University, Kaohsiung, Taiwan; 2 Department of Medical Laboratory Science, College of Medical Science and Technology, I-Shou University, Kaohsiung, Taiwan; 3 Department of Bioscience and Biotechnology, National Taiwan Ocean University, Keelung, Taiwan; Innovations Therapeutiques et Resistances (INTHERES), Université de Toulouse, Toulouse, France

**Keywords:** *Mycobacterium abscessus*, *Mycobacterium fortuitum*, *Mycobacterium chelonae*, antibiotic resistance, biofilm, naturally occurring compounds, synergy

## Abstract

Some naturally occurring compounds, known for their antimicrobial activities, have been employed as food additives. However, their efficacy in treating infections caused by antibiotic-resistant bacteria is yet to be fully explored. Rapidly growing mycobacteria (RGM), a category within nontuberculous mycobacteria (NTM), are prevalent in various environments and can lead to infections in humans. The rise of antimicrobial resistance within RGM is a documented concern. In this study, we reported that four specific natural compounds effectively inhibited the growth and biofilm formation of three key RGM pathogens *M. abscessus*, *M. fortuitum*, and *M. chelonae*. We screened 12 natural compounds for their effectiveness against antibiotic-resistant clinical strains of RGM. Four compounds showed significant inhibitory effects from the most effective to least: *trans*-cinnamaldehyde, carvacrol, gentisaldehyde, and phloroglucinaldehyde. In the analysis of time-killing kinetics, gentisaldehyde and phloroglucinaldehyde displayed bactericidal activity while *trans*-cinnamaldehyde and carvacrol exhibited bacteriostatic effects. At 1× minimal inhibition concentrations, these compounds significantly reduced biofilm formation in all three RGM species to levels between 2.9% and 20.5% relative to controls. Checkerboard assays indicated synergistic interactions between these four compounds and antibiotics such as amikacin, clarithromycin, and linezolid. Of these 12 compound-antibiotic combinations, the pairs of carvacrol-linezolid, carvacrol-amikacin, and gentisaldehyde-clarithromycin demonstrated the most synergy against multiple RGM strains. Moreover, two other compounds citral and geraniol showed synergism with all three test antibiotics. Time-killing assays further confirmed most of synergistic combinations identified in the checkerboard tests. Our research suggests the potential of these essential oils and phenolic aldehydes, both individually and in combination with antibiotics, in treating RGM infections. In addition, this work illuminates applications of these natural compounds in environmental remediation to mitigate bacterial persistence for the control of infectious diseases.

**IMPORTANCE:**

The emergence of antimicrobial resistance within rapidly growing mycobacteria (RGM) poses a significant threat to public health. This study investigates the potential of naturally occurring compounds to combat infections caused by antibiotic-resistant RGM including *M. abscessus*, *M. fortuitum*, and *M. chelonae*. We identified four specific natural compounds showing impressive inhibitory effects against antibiotic-resistant clinical strains. These compounds not only inhibited the growth and biofilm formation but also exhibited synergistic interactions with antibiotics against key RGM pathogens. Our findings highlight the alternative treatment strategies for RGM infections and potential environmental applications of these natural compounds in mitigating microbial persistence and controlling infectious diseases.

## INTRODUCTION

Mycobacteria are important human pathogens and cause a variety of diseases. Nontuberculous mycobacteria (NTM) are usually opportunistic pathogens whose role in human disease is increasingly recognized, especially regarding the rapidly growing mycobacteria (RGM) (less than 7 days to form visible colonies on solid media) (1, 2). RGM include a diverse group of species and are ubiquitous in both natural (such as soil and water) and man-made environments (such as water systems) (2). They can account for many mycobacteriosis occurrences and have high lethal power in immunocompromised patients (3). RGM have been a clinical concern because they can cause a wide spectrum of infections in the lungs, skin, soft tissues, blood, and other parts of the body (2). In some countries, RGM have become the second most common NTM recovered from respiratory specimens (4, 5).

Among RGM, *M. abscessus*, *M. fortuitum*, and *M. chelonae* are the most important species and often associated with human diseases (1, 2, 6, 7). *M. abscessus* is an emerging pathogen and the most common cause of lung disease among RGM (8). It is responsible for severe respiratory, skin, and mucosal infections. It is often regarded as one of the most antibiotic-resistant mycobacteria (8). *M. fortuitum* is the most common RGM to cause extrapulmonary diseases and mainly causes skin and bone/joint infections (7). *M. chelonae*, commonly associated with skin and soft tissue infections, can cause catheter-related infections and post-surgical infections after implants, transplants, and injections (1, 7). RGM has gained increasing clinical importance since an increment of infections was observed (1, 9–11).

High prevalence of antimicrobial resistance in RGM has been reported (6, 12–14). Clinically, *M. abscessus* and *M. chelonae* isolates are often more resistant to antibiotics than other RGM species (2). Due to diverse drug resistance, treatment against RGM becomes challenging and can be lengthy and expensive (6, 15). An important pathogenic factor of mycobacteria is the formation of biofilms, which is often associated with antimicrobial resistance of mycobacteria (16). Due to the high hydrophobicity, RGM and other NTM favor the formation of biofilms, accounting for their resistance to antibiotics and commonly used disinfectants (16, 17). RGM can form biofilm structures in medical and environmental settings, such as biomedical devices, water distribution systems, cosmetic surgery, and catheters, which could contribute to therapy failure and relapses (16, 18). With increasing awareness of the clinical importance of RGM, it is necessary to explore and develop new strategies to block biofilm formation as an important cause of human infection.

The global emergence of antimicrobial resistance (AMR) in bacteria has raised the interests to seek natural sources for alternative or adjunct antimicrobial agents to control microbial pathogens. The antimicrobial activities of naturally occurring compounds, such as essential oils (EO) and phenolic compounds, are increasingly recognized (19–21). These compounds are usually plant secondary metabolites, which can be obtained naturally from various parts of plant materials or be synthesized. Some antimicrobial natural compounds are used in food industry against food-borne pathogens since they own the preservative potency and are generally “regarded as safe.” Nevertheless, the therapeutic potency of natural compounds against clinically important pathogens especially against antibiotic-resistant isolates remains to be clarified. In addition, combinations of phytochemicals with antibiotics to enhance the efficacy of antibiotics have been considered as one promising strategy to fight resistant pathogens (22). Synergism of some plant natural products combined with antibiotics has been observed and several essential oils have been suggested to potentiate existing antibiotics or prolong the lifespan of existing antibiotics (22, 23).

In this work, we describe our findings from testing the antimicrobial activities of various naturally occurring compounds against RGM clinical isolates. By screening of 12 antimicrobial candidates, we identified 6 anti-RGM compounds, which inhibited three clinically important species. Minimal inhibition concentration (MICs) and time-killing kinetics of compounds against RGM were compared. We demonstrated that several compounds reduced the biofilm formation of RGM and synergistically interacted with antibiotics against drug-resistant strains.

## RESULTS

### Naturally occurring compounds inhibit antibiotic-resistant *M. abscessus*, *M. chelonae*, and *M. fortuitum*

To identify the naturally occurring compounds with inhibitory activities on RGM, we initially tested 12 commercially available compounds against clinical strains of RGM, including three major species *M. abscessus*, *M. chelonae*, and *M. fortuitum*. Tested RGM strains showed a wide range of antibiotic resistance (Table 1). In disk diffusion assays, we observed that six compounds induced inhibition zones upon all tested RGM strains (Table 2; Fig. S1), including *trans*-cinnamaldehyde, carvacrol, citral, geraniol, gentisaldehyde, and phloroglucinaldehyde. The other compounds, including capsaicin, caffeic acid, chlorogenic acid, vanillic acid, berberine chloride, and palmatine chloride, did not cause the formation of inhibition zones or only caused very small zones (not shown). The structures of six identified anti-RGM compounds are shown in Fig. S2. *Trans*-cinnamaldehyde, gentisaldehyde, and phloroglucinaldehyde belong to aldehydes. Carvacrol, citral, and geraniol belong to monoterpenes. *Trans*-cinnamaldehyde, carvacrol, citral, and geraniol, which can be found in many essential oils, have been approved to be used as food additives or flavoring agents by the Food and Agriculture Organization of the United Nations (FAO) and World Health Organization (WHO) (24). Gentisaldehyde and phloroglucinaldehyde are not commonly used as food additives. Acute toxicity information about LD_50_ (lethal dose, 50%) values of these anti-RGM compounds (except gentisaldehyde) are listed in Table S1, in the range of 810–4,960 mg/kg (oral). LD_50_ of gentisaldehyde was unknown, but its cytotoxicity was suggested low (25).

**TABLE 1:** Antibiotics resistance profiles of RGM clinical strains in this study^*a*^

Strains	Species	SXT	LZD	CIP	IMI	MXF	FOX	AMI	DOX	MIN	TOB	CLA
MAB1	*M. abscessus*	R	R	R	I	R	R	S	R	R	R	R
MAB4	*M. abscessus*	R	R	R	R	R	I	S	R	R	R	I
MAB5	*M. abscessus*	R	R	R	I	R	R	S	R	R	R	R
MC4	*M. chelonae*	S	R	S	R	S	R	S	I	S	R	R
MC6	*M. chelonae*	R	S	S	I	S	R	S	R	R	S	S
MC7	*M. chelonae*	R	I	R	R	R	R	S	R	R	S	S
MF1	*M. fortuitum*	R	S	S	I	S	R	R	R	R	R	R
MF3	*M. fortuitum*	R	R	R	I	S	I	S	R	R	R	R
MF6	*M. fortuitum*	R	R	S	R	S	R	S	R	R	R	R

^*a*^ Determined using broth microdilution-based automated antimicrobial susceptibility testing (AST) system SENSITITRE RAPMYCOI (TREK/Thermo Scientific). S: Susceptible; I: Intermediate; R: Resistant. Antibiotics: SXT, trimethoprim/sulfamethoxazole; LZD, linezolid; CIP, ciprofloxacin; IMI, imipenem; MXF, moxifloxacin; FOX, cefoxitin; AMI, amikacin; DOX, doxycycline; MIN, minocycline; TOB, tobramycin; CLA, clarithromycin.

**TABLE 2:** Inhibition zone sizes (mm) by naturally occurring compounds against RGM strains in the disk diffusion assays^*a*^

Strains	tCIN	CAR	CIT	GER	GEN	PHL	AMI
*M. abscessus*							
MAB1	43	14	40	28	19	16	24
MAB4	33	16	40	40	17	15	23
MAB5	35	16	L	36	18	15	25
*M. chelonae*							
MC4	55	12	L	32	32	29	40
MC6	L	L	41	L	21	32	29
MC7	L	14	L	L	22	22	16
*M. fortuitum*							
MF1	30	14	22	20	17	19	16
MF3	54	13	27	21	21	22	20
MF6	15	13	30	26	17	21	21
*M. smegmatis*							
ATCC 14468	42	44	44	26	16	18	30

^*a*^ tCIN, *trans*-cinnamaldehyde (99 mg/mL); CAR, carvacrol (39.2 mg/mL); CIT, citral (95 mg/mL); GER, geraniol (98 mg/mL); GEN, gentisaldehyde (50 mg/mL); PHL, phloroglucinaldehyde (50 mg/mL); AMI, amikacin (3 mg/mL). Liquid compounds were tested for their highest concentrations and solid compounds were tested for the stock solutions. “L” indicates the inhibition zone size larger than 60 mm. “-” indicates no inhibition. Paper disk diameter 8 mm; test volume 20 μL. *M. smegmatis* ATCC 14468 was a non-pathogenic RGM reference strain to test for comparison.

### MICs) of natural occurring compounds against RGM

The MIC values of six identified anti-RGM compounds were determined (Table 3). The tested six compounds showed MICs ranging from 32 to 1,024 µg/mL. Against *M. abscessus*, the most effective compound was *trans*-cinnamaldehyde (MIC 64 µg/mL), followed by carvacrol (MIC 128 µg/mL) and gentisaldehyde (MIC 256 µg/mL). Against *M. chelonae*, the most effective compounds were carvacrol (MIC 32–64 μg/mL) and *trans*-cinnamaldehyde (MIC 32–128 μg/mL), followed by gentisaldehyde (MIC 256 µg/ml) and phloroglucinaldehyde (MIC 256–512 μg/mL). Against *M. fortuitum*, the most effective compounds were *trans*-cinnamaldehyde (MIC 64 µg/mL) and carvacrol (MIC 64–128 μg/mL), followed by gentisaldehyde (MIC 256 µg/mL) and phloroglucinaldehyde (MIC 512 µg/mL). Overall, among the six anti-RGM compounds, *trans*-cinnamaldehyde and carvacrol were the most effective, followed by gentisaldehyde and phloroglucinaldehyde.

**TABLE 3:** MIC values (μg/mL) of naturally occurring compounds against RGM strains^*a*^

Strains	Species	tCIN	CAR	CIT	GER	GEN	PHL	AMI
MAB1	*M. abscessus*	64	128	512	1,024	256	512	4
MAB	*M. abscessus*	64	128	512	1,024	256	512	16
MAB5	*M. abscessus*	64	128	512	1,024	256	512	8
MC4	*M. chelonae*	128	64	1024	1,024	256	512	2
MC6	*M. chelonae*	128	64	1024	1,024	256	256	4
MC7	*M. chelonae*	32	32	1024	1,024	256	256	8
MF1	*M. fortuitum*	64	64	1024	1,024	256	512	64
MF3	*M. fortuitum*	64	128	1024	1,024	256	512	8
MF6	*M. fortuitum*	64	64	1024	1,024	256	512	4
ATCC14468	*M. smegmatis*	128	128	512	1,024	256	512	4

^*a*^ Determined using broth microdilution assays. tCIN, *trans*-cinnamaldehyde; CAR, carvacrol; CIT, citral; GER, geraniol; GEN, gentisaldehyde; PHL, phloroglucinaldehyde; AMI, amikacin. RGM reference strain *M. smegmatis* ATCC14468 was analyzed for comparison.

### Time-killing kinetics of anti-RGM compounds

**Fig 1:**
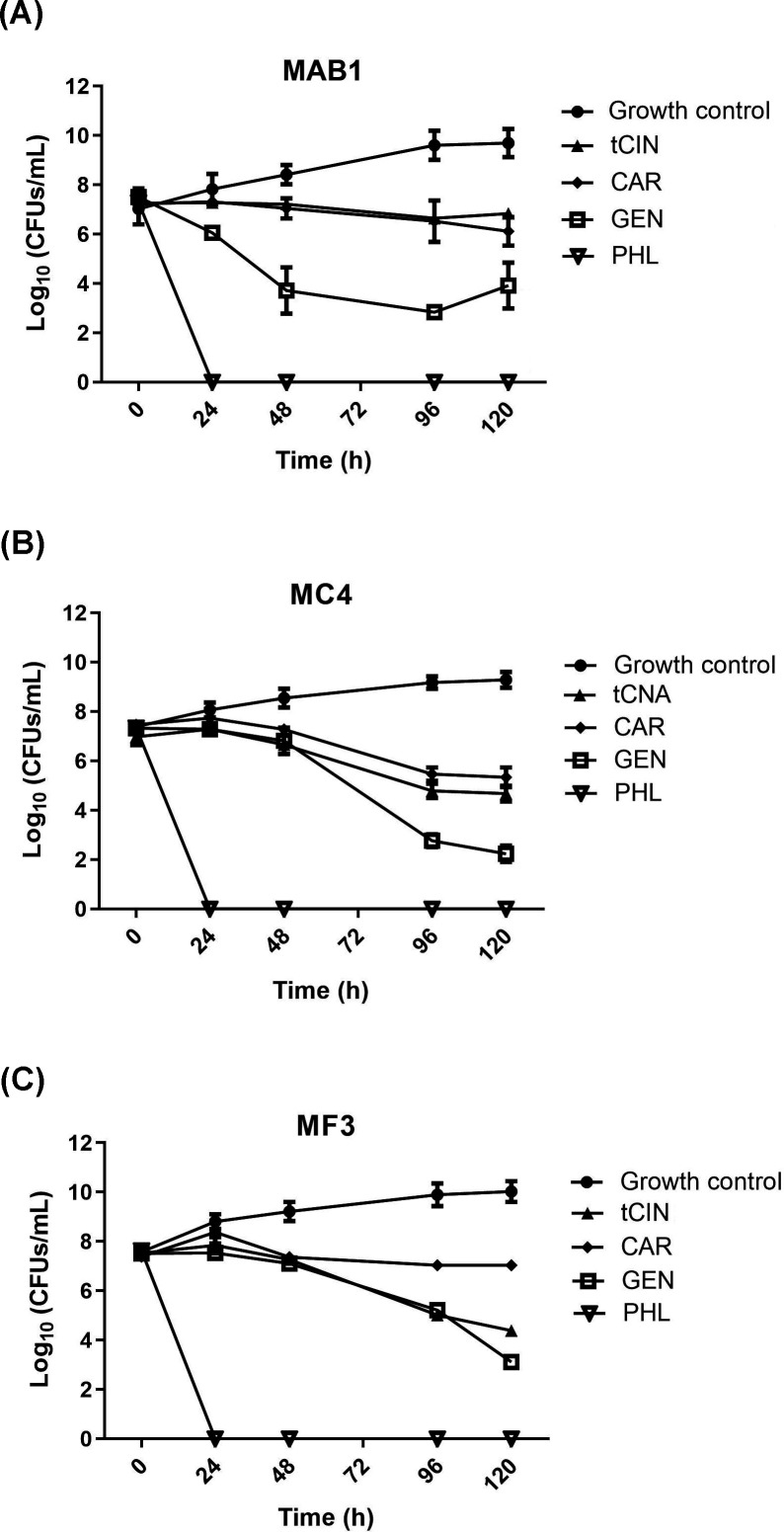
Time-killing kinectics of anti-RGM compounds. *trans*-Cinnamaldehyde (tCIN), carvacrol (CAR), gentisaldehyde (GEN), and phloroglucinaldehyde (PHL) were tested at 1× MIC concentrations against three RGM species: (**A**) *M. abscessus* MAB1, (**B**) *M. chelonae* MC4, (**C**) *M. fortuitum* MF3.

The time-kill kinetics profiles of *trans*-cinnamaldehyde, carvacrol, gentisaldehyde, and phloroglucinaldehyde were further characterized (Fig. 1). Compared to the growth control curves which exhibited a gradual rise up, these four compounds against three RGM species all caused a reduction in the number of viable cells between 48 h and 120 h. While *trans*-cinnamaldehyde and carvacrol appeared to be bacteriostatic within 120 h, gentisaldehyde and phloroglucinaldehyde showed bactericidal effects. Gentisaldehyde exhibited the bactericidal activity against *M. abscessus* MAB1 between 48 h and 120 h, *M. chelonae* MC4 between 96 h and 120 h, and *M. fortuitum* MF3 at 120 h. Phloroglucinaldehyde exhibited notable bactericidal activities against three test RGM strains between 24 h and 120 h.

### Inhibition of RGM biofilm by *trans*-cinnamaldehyde, carvacrol, gentisaldehyde, and phloroglucinaldehyde

**Fig 2:**
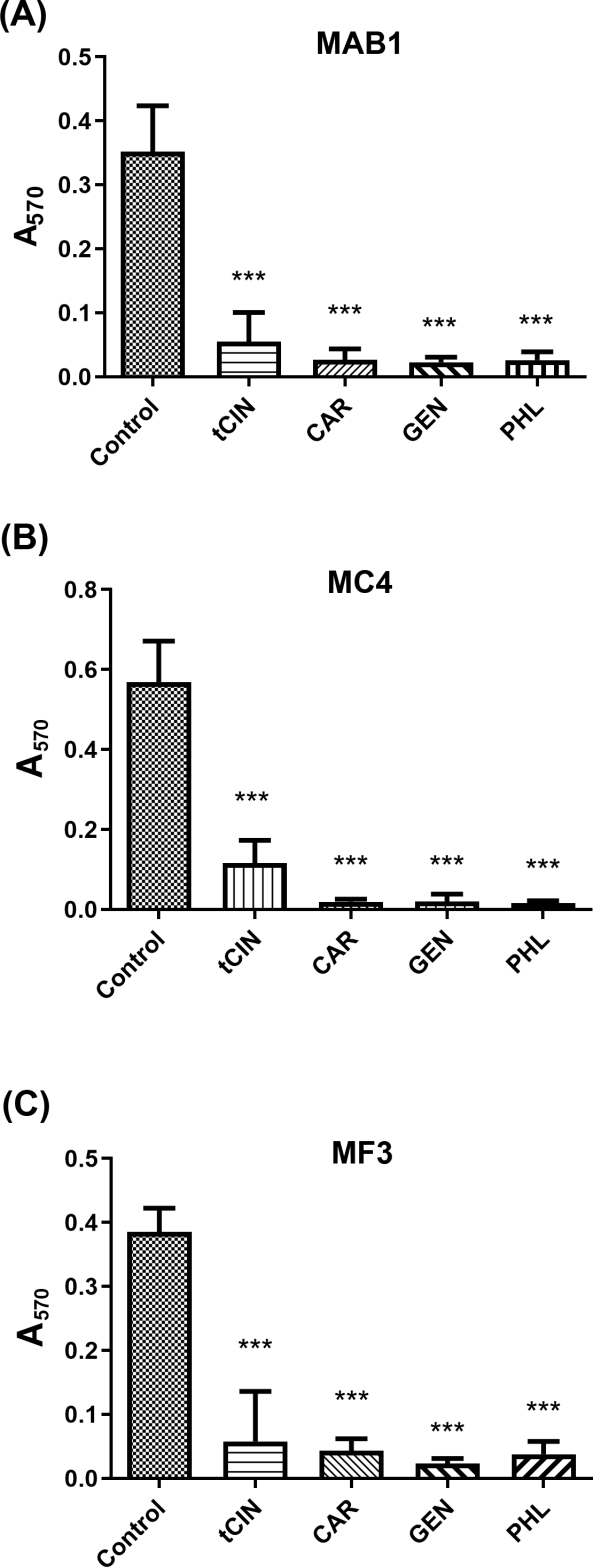
Compound effects on biofilm formation of RGM: (**A**) *M. abscessus* MAB1, (**B**) *M. chelonae* MC4, and (**C**) *M. fortuitum* MF3. *trans*-Cinnamaldehyde (tCIN), carvacrol (CAR), gentisaldehyde (GEN), and phloroglucinaldehyde (PHL) were tested at 1× MIC concentrations. RGM biofilm levels were determined using crystal violet-based biofilm assays, quantified spectrophotometrically at 570 nm. Each bar indicates the mean ± SEM from three independent experiments. *P < 0.05, compared to control, analysis of variance followed by Bonferroni multiple comparisons test.

Biofilm formation is a successful survival strategy for RGM, which could be difficult to eradicate with common decontamination practices (16, 17). We assessed the compound effects on biofilm formation of RGM (Fig. 2). In the biofilm assays, we observed that *trans*-cinnamaldehyde, carvacrol, gentisaldehyde, and phloroglucinaldehyde significantly inhibited the biofilm formation of RGM clinical strains *M. abscessus* MAB1, *M. chelonae* MC4, and *M. fortuitum* MF3. Against three different strains, *trans*-cinnamaldehyde, carvacrol, gentisaldehyde, and phloroglucinaldehyde at 1× MIC concentration reduced RGM biofilm to 14.9%–20.5%, 3.4%–11.2%, 3.5%–6.4%, and 2.9%–9.8%, respectively, compared to the control.

### Identification of synergistic interactions between anti-RGM compounds and antibiotics

We further assessed the potential synergistic interactions between naturally occurring compounds and antibiotics. We conducted checkerboard dilution tests to analyze the combinations of six natural compounds (*trans*-cinnamaldehyde, carvacrol, gentisaldehyde, phloroglucinaldehyde, citral, and geraniol) with three antibiotics (amikacin, clarithromycin, and linezolid) against 10 RGM strains (Table 4). Among a total of 18 tested compound-antibiotic combinations, synergistic interactions were observed in 16 combinations (against at least one RGM strain). Nine combinations showed synergism (SY) against more than half of the test strains (>50%): carvacrol-amikacin (6 out of 10 strains, 60%), carvacrol-linezolid (7 out of 10 strains, 70%), gentisaldehyde-clarithromycin (6 out of 10 strains, 60%), citral-amikacin (9 out of 10 strains, 90%), citral-clarithromycin (8 out of 10 strains, 80%), citral-linezolid (6 out of 10 strains, 60%), geraniol-amikacin (9 out of 10 strains, 90%), geraniol-clarithromycin (8 out of 10 strains, 80%), and geraniol-linezolid (6 out of 10 strains, 60%). The combinations exhibiting synergism against the largest number of RGM strains were citral-amikacin and geraniol-amikacin. *Trans*-cinnamaldehyde, carvacrol, citral, and geraniol demonstrated synergistic interactions with all three test antibiotics. Gentisaldehyde and phloroglucinaldehyde only exhibited synergistic effects with clarithromycin and linezolid. When combined with amikacin, gentisaldehyde and phloroglucinaldehyde displayed addictive or indifferent effects against all tested RGM strains. None of the compound-antibiotic combinations demonstrated antagonistic effects.

**TABLE 4:** Synergistic interactions of natural compounds in combination with antibiotics against RGM strains^*a*^

		Antibiotics					
Natural		AMI		CLA		LZD	
Compounds	Strain	FICI	Interpretation	FICI	Interpretation	FICI	Interpretation
tCIN	MAB1	0.50	SY	0.63	AD	0.38	SY
	MAB4	0.38	SY	0.75	AD	0.52	AD
	MAB5	0.38	AD	0.28	SY	0.50	SY
	MC4	0.53	AD	0.28	SY	0.38	SY
	MC6	1.00	AD	1.00	AD	0.53	AD
	MC7	0.75	AD	0.75	AD	0.63	AD
	MF1	0.38	SY	0.63	AD	1.25	IN
	MF3	0.25	SY	0.25	SY	2.03	IN
	MF6	0.38	SY	0.38	SY	2.06	IN
	ATCC14468	0.53	AD	0.27	SY	0.25	SY
CAR	MAB1	2.06	AD	0.38	SY	0.19	SY
	MAB4	0.38	SY	0.75	AD	0.53	AD
	MAB5	0.38	SY	0.50	SY	0.27	SY
	MC4	0.75	AD	0.63	AD	0.50	SY
	MC6	1.00	AD	0.63	AD	0.38	SY
	MC7	0.75	AD	0.75	AD	0.56	AD
	MF1	0.50	SY	4.01	IN	1.00	AD
	MF3	0.28	SY	1.02	AD	0.19	SY
	MF6	0.50	SY	1.50	IN	0.50	SY
	ATCC14468	0.50	SY	0.31	SY	0.25	SY
GEN	MAB1	1.03	AD	0.38	SY	1.25	IN
	MAB4	0.53	AD	0.53	AD	1.25	IN
	MAB5	1.03	AD	0.38	SY	1.25	IN
	MC4	0.63	AD	0.31	SY	1.03	AD
	MC6	0.56	AD	0.53	AD	0.38	SY
	MC7	0.56	AD	0.56	AD	0.56	AD
	MF1	0.56	AD	0.52	AD	1.03	AD
	MF3	0.53	AD	0.38	SY	0.75	AD
	MF6	0.53	AD	0.50	SY	0.75	AD
	ATCC14468	0.75	AD	0.27	SY	0.75	AD
PHL	MAB1	1.02	AD	0.63	AD	1.00	AD
	MAB4	1.02	AD	0.52	AD	1.00	AD
	MAB5	1.02	AD	0.38	SY	1.00	AD
	MC4	1.02	AD	0.56	AD	0.75	AD
	MC6	1.03	AD	2.03	IN	0.38	SY
	MC7	1.13	IN	0.53	AD	0.50	SY
	MF1	0.52	AD	0.51	AD	1.00	AD
	MF3	0.52	AD	0.56	AD	1.00	AD
	MF6	0.52	AD	0.56	AD	0.63	AD
	ATCC14468	1.25	IN	0.75	AD	1.50	IN
CIT	MAB1	0.50	SY	0.31	SY	0.63	AD
	MAB4	0.50	SY	0.38	SY	0.50	SY
	MAB5	0.38	SY	0.25	SY	0.38	SY
	MC4	0.57	AD	0.53	AD	1.27	IN
	MC6	0.38	SY	0.50	SY	1.00	AD
	MC7	0.19	SY	0.25	SY	0.32	SY
	MF1	0.08	SY	0.56	AD	0.50	SY
	MF3	0.28	SY	0.38	SY	0.33	SY
	MF6	0.13	SY	0.25	SY	0.38	SY
	ATCC14468	0.16	SY	0.38	SY	0.75	AD
GER	MAB1	0.50	SY	0.13	SY	0.19	SY
	MAB4	0.19	SY	0.19	SY	0.19	SY
	MAB5	0.25	SY	0.31	SY	0.25	SY
	MC4	0.39	SY	1.02	IN	0.63	AD
	MC6	0.56	AD	2.00	IN	1.13	IN
	MC7	0.19	SY	0.16	SY	0.19	SY
	MF1	0.19	SY	0.25	SY	0.75	AD
	MF3	0.19	SY	0.13	SY	0.13	SY
	MF6	0.13	SY	0.16	SY	0.16	SY
	ATCC14468	0.13	SY	0.09	SY	0.63	AD

^*a*^ Determined using the checkerboard assays. Fractional inhibitory concentration Indexes (FICIs) were interpreted as synergistic (SY) when it was ≦0.5, as additive (AD) when it was >0.5–1, indifferent (IN) when it was >1–4.0, and antagonistic (AN) when it was >4 (Phytomedicine. 2015). tCIN, *trans*-Cinnamaldehyde; CAR, carvacrol; GEN, gentisaldehyde; PHL, phloroglucinaldehyde; CIT, citral; GER, geraniol; AMI, amikacin; CLA, clarithromycin; LZD, linezolid.

**Fig 3:**
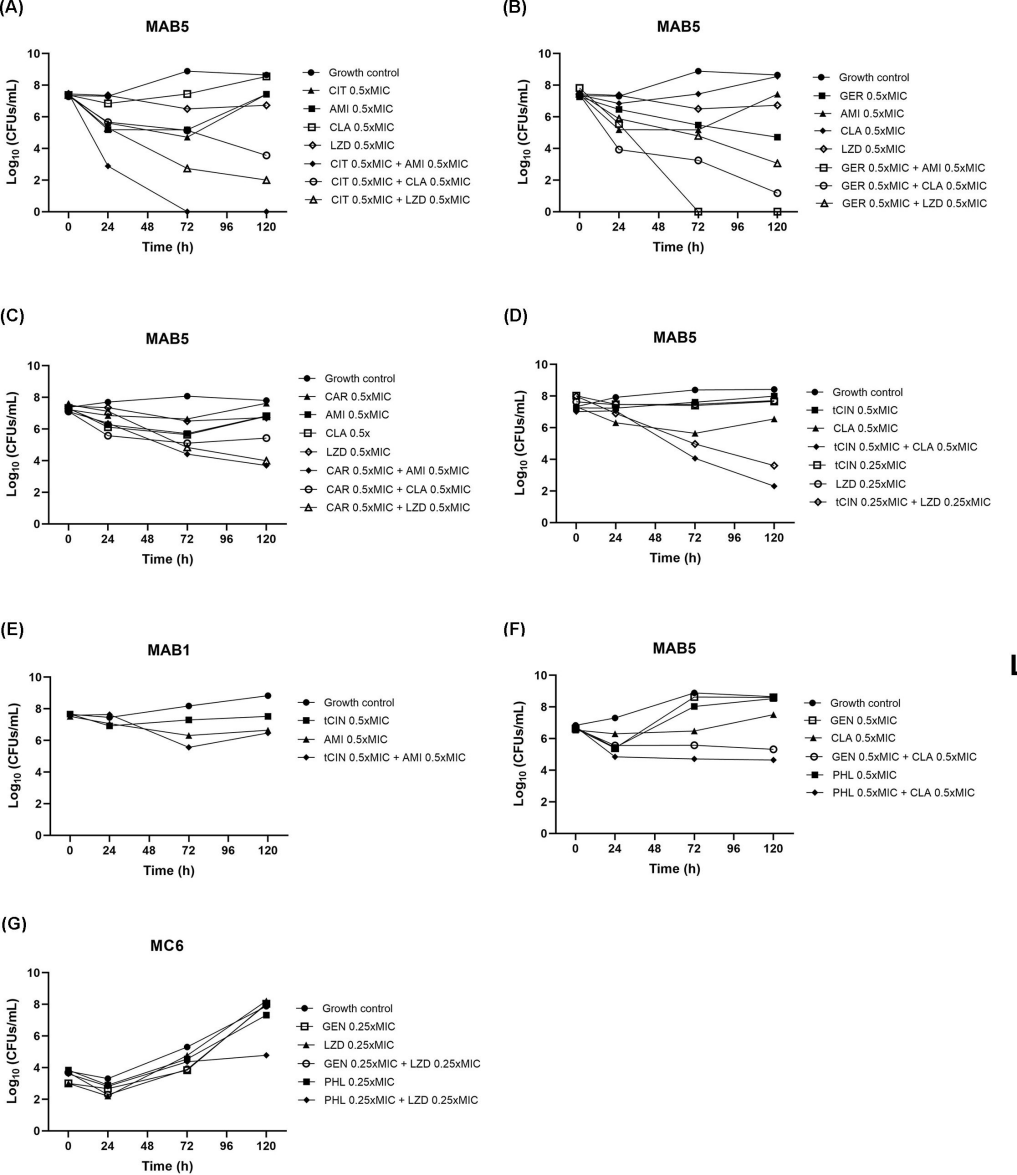
Time-killing analysis to check synergistic combinations of anti-RGM compounds and antibiotics. Sixteen compound-antibiotic combinations were tested against selected RGM strain MAB5, MAB1, or MC6 based on the results of checkerboard assays: (**A**) Citral (CIT) combined with amikacin (AMI), clarithromycin (CLA), or linezolid (LZD); (**B**) geraniol (GER) combined with AMI, CLA, or LZD; (**C**) carvacrol (CAR) combined with AMI, CLA, or LZD; (**D**) *trans*-cinnamaldehyde (tCIN) combined with AMI, CLA, or (**E**) with LZD; (**F**) gentisaldehyde (GEN) or phloroglucinaldehyde (PHL) combined with CLA; (**G**) GEN or PHL combined with LZD.

Time-kill assays were further conducted to evaluate 16 synergistic combinations identified in the above checkerboard assays (Fig. 3A through G). Twelve of the sixteen compound-antibiotic combinations showed synergistic interplay against RGM, by revealing a ≧2 log_10_ decrease in CFUs/mL (compared with the most active single agent): citral combined with amikacin, clarithromycin, or linezolid (Fig. 3A); geraniol combined with amikacin or clarithromycin (Fig. 3B); carvacrol combined with amikacin or linezolid (Fig. 3C); *trans*-cinnamaldehyde combined with clarithromycin or linezolid (Fig. 3D); gentisaldehyde combined with clarithromycin (Fig. 3F); and phloroglucinaldehyde combined with clarithromycin (Fig. 3F) or linezolid (Fig. 3G). Strong synergistic interactions by causing ≧3 log_10_ reduction (26) were observed in citral-amikacin, citral-clarithromycin, citral-linezolid, and geraniol-amikacin combinations.

## DISCUSSION

The exploration of antimicrobial activity from natural compounds presents a novel strategy to address the antimicrobial resistance (AMR) issue prevalent in microorganisms of mycobacteria. Among the Nontuberculous mycobacteria (NTM), rapidly growing mycobacteria (RGM) are of particular concern due to their high prevalence of antibiotic resistance. In this study, we screened 12 naturally occurring antimicrobial compounds and identified six—*trans*-cinnamaldehyde, carvacrol, citral, geraniol, gentisaldehyde, and phloroglucinaldehyde—that inhibited RGM strains, including three major clinical species: *M. abscessus*, *M. chelonae*, and *M. fortuitum*. Among these six anti-RGM compounds, *trans*-cinnamaldehyde and carvacrol proved to be the most effective, followed by gentisaldehyde and phloroglucinaldehyde. Through clinical isolate testing, this study underscores the potential of plant essential oils and phenolic components in inhibiting antibiotic-resistant mycobacteria.

We noted four common compounds in essential oils—*trans*-cinnamaldehyde, carvacrol, citral, and geraniol—that inhibited antibiotic-resistant RGM. In particular, *trans*-cinnamaldehyde and carvacrol exhibited the lowest MIC values, thus signifying the highest level of activity against RGM strains. These two essential oils have previously been shown to inhibit a variety of bacterial species (27–33). To our knowledge, inhibitory effects of *trans*-cinnamaldehyde on clinical RGM including *M. abscessus*, *M. chelonae*, and *M. fortuitum* were described for the first time in the present study. Cinnamaldehyde, a phenylpropene aldehyde, was originally isolated from cinnamon essential oil (28). Its antimicrobial activity has been reported against a diverse range of bacteria, such as *E. coli, Bacillus subtilis, Staphylococcus spp., Listeria spp.* and *Salmonella spp., Lactobacillus sakei, Campylobacter jejuni, Vibrio* spp., *Pseudomonas* spp., *Klebsiella pneuminiae, Porphyromonas gingivalis*, *Streptococcus pyogenes*, and *Cronobacter sakazakii,* etc. (28, 29). Regarding mycobacteria, cinnamaldehyde has shown inhibitory effects on *M. tuberculosis* (30) and *M. avium* subsp. *paratuberculosis* (27). The potential mechanisms through which cinnamaldehyde inhibits bacteria include damaging the cell membrane (34), altering lipid profiles (35), inhibiting ATPases (36), or obstructing cell division (37). Carvacrol, a phenolic monoterpene, is a major component of essential oils from the *Labiatae* plant family (38). It has demonstrated inhibitory effects on a wide array of bacteria including food-borne bacteria and drug-resistant pathogens like *Bacillus cereus*, *Enterococcus faecalis*, *Listeria monocytogenes*, *Staphylococcus aureus,* and *E. coli,* to name a few (31, 32, 39). In mycobacteria, carvacrol’s inhibitory actions were noted against *M. tuberculosis* (33), *M. avium* subsp. *paratuberculosis* (27), and RGM species *M. abscessus*, *M. chelonae*, and *M. fortuitum* in a recent parallel study (40). The potential antimicrobial mechanisms of carvacrol may involve its ability to permeabilize and depolarize the cytoplasmic membrane, resulting in bacterial membrane damage (39). Given their noteworthy antimicrobial activities against RGM, both *trans*-cinnamaldehyde and carvacrol warrant further *in vivo* studies to evaluate their efficacy in treating mycobacterial infections.

This study spotlighted two phenolic benzaldehydes, gentisaldehyde, and phloroglucinaldehyde, exhibiting bactericidal activities against clinical RGM species. Unlike cinnamaldehyde and carvacrol, there is a scant amount of detailed information regarding the antimicrobial activities and mechanisms of gentisaldehyde and phloroglucinaldehyde. Gentisaldehyde and phloroglucinaldehyde, also known as 2,5-dihydroxybenzaldehyde and 2,4,6-trihydroxybenzaldehyde, are primarily identified as secondary metabolites in plants (23). Our present study is the pioneer in unveiling the anti-mycobacterial activity of phloroglucinaldehyde, as well as demonstrating the effects of gentisaldehyde against RGM. Our findings revealed that both benzaldehydes inhibited RGM, albeit with varying antimicrobial effectiveness and kinetics. Against most RGM strains, gentisaldehyde exhibited lower MIC values (256 µg/mL) as compared to phloroglucinaldehyde (512 µg/mL). However, phloroglucinaldehyde manifested an earlier bactericidal effect against RGM (at 24 h) in the kinetic curves. A screening of 35 benzaldehydes identified gentisaldehyde and phloroglucinaldehyde as having the highest antimicrobial activities against four food-borne pathogens (testing one strain of each species) including *Campylobacter jejuni*, *E. coli* O157:H7, *L. monocytogenes*, and *Salmonella enterica* (23). However, an assessment of 18 natural compounds against *M. avium* subsp. *paratuberculosis* (a slowly growing mycobacteria species) reported inhibition by gentisaldehyde but not by phloroglucinaldehyde (27). Combined with our findings, it is suggested that diverse benzaldehydes exhibit varying inhibitory effects against different bacterial species. Further investigations into the antimicrobial spectrum and potential mechanisms of gentisaldehyde and phloroglucinaldehyde, especially against different mycobacterial species, are warranted.

RGM are ubiquitous environmental microorganisms found in diverse habitats and recovered from various water sources, including biofilms in plumbing systems (19, 31). Bacterial biofilms are often implicated in device-related infections and enhanced bacterial resistance to antimicrobials. Our study illustrated that *trans*-cinnamaldehyde, carvacrol, gentisaldehyde, and phloroglucinaldehyde significantly inhibit the biofilm formation of RGM strains. Hence, contemplating the use of these natural compounds for environmental sanitation and remediation, such as in hospital environments, sinks, faucets, or water systems, is meritorious. Employing these natural compounds to target RGM biofilm can be envisaged as a potential strategy to curtail mycobacterial transmission and infections in forthcoming times.

The emergence of drug resistance in nontuberculous mycobacteria poses serious clinical challenges, particularly among immunocompromised patients (7). Employing combinations of agents may serve as one strategy to address the issue of resistance. This current study disclosed that *trans*-cinnamaldehyde, carvacrol, gentisaldehyde, phloroglucinaldehyde, citral, and geraniol interacted synergistically with antibiotics against clinical RGM. To our knowledge, the synergistic interaction of gentisaldehyde and phloroglucinaldehyde with antibiotics has been reported for the first time through this study. In checkerboard assays, we observed that gentisaldehyde and phloroglucinaldehyde synergistically interacted with clarithromycin and linezolid against RGM. Carvacrol, *trans*-cinnamaldehyde, citral, and geraniol synergistically interacted with all three test antibiotics against RGM. Carvacrol and *trans*-cinnamaldehyde have been suggested as antibiotic potentiators to enhance the antimicrobial activities of other antibiotics (41). The synergistic activities of citral and geraniol in combination with antibiotics against mycobacteria were demonstrated for the first time in the present study. The three antibiotics evaluated against RGM in our study possess human toxicity: amikacin is known for its nephrotoxicity (which damages the kidneys) and ototoxicity (which can lead to hearing loss) (42); linezolid exhibits mitochondrial toxicity and may induce bone marrow suppression, lactic acidosis, and neuropathy (43); clarithromycin, while relatively less toxic, may cause gastrointestinal side effects (44). Therefore, the naturally occurring compounds identified in the present study could be considered for use in combination with these antibiotics to enhance efficacy and to potentially reduce the dosage required to combat infections by *M. abscessus*, *M. chelonae*, and *M. fortuitum*.

Most SY combinations in the checkerboard assays were confirmed as synergism using time-killing assays (12 out of 16). Several factors could cause the different results between the checkerboard and time-killing assays. For example, these two methods utilize different conditions, such as inoculum size and culture volume, and measure different phenomena (inhibitory activity vs bactericidal activity). The checkerboard method is relatively easy to perform and suitable for screening experiments to identify potential combinations. Time-killing analysis is more time-consuming and labor-intensive, and can only test one concentration or one ratio of the antimicrobials at one time to establish one time-killing curve. The test often needs to be repeated to observe interactions at other concentrations and ratios. Since most synergistic combinations against RGM in this study were confirmed by both methods, these combinations are worth further investigation of *in vivo* synergy in the future.

In summary, the current study demonstrates the anti-mycobacterial, anti-biofilm, and antibiotic synergistic activities against RGM clinical strains through the screening of 12 compounds. Specifically, *trans*-cinnamaldehyde, carvacrol, gentisaldehyde, and phloroglucinaldehyde emerged as the most effective candidates to inhibit drug-resistant RGM. The findings advocate for the applications of these natural compounds in environmental remediation to mitigate bacterial persistence and hence reduce the risk of infection. This study also broadens the understanding regarding the potential use of natural compounds either alone or in tandem with antibiotics to treat RGM infections.

## MATERIALS AND METHODS

### Test compounds

Twelve compounds were obtained from Sigma-Aldrich (St. Louis, MO, USA): *trans*-cinnamaldehyde (C80687, 99% purity), carvacrol (282197, 98% purity), citral (C83007, 95% purity), geraniol (163333, 98% purity), gentisaldehyde (D108200), phloroglucinaldehyde (T65404), capsaicin (360376), caffeic acid (C0625), chlorogenic acid (C3878), vanillic acid (H36001), berberine chloride (B3251), and palmatine chloride (361615). Purchased essential oil *trans*-cinnamaldehyde, carvacrol, citral, and geraniol were in the liquid form. The others were solid compounds, of which the stock solutions (50 mg/mL) were prepared by suspending each compound in absolute ethanol. The final concentration of ethanol present in the growth medium was standardized at 0.4% (27). Ethanol 0.4% was preliminarily tested and did not inhibit the growth of RGM strains (data not shown).

### Bacterial strains

Clinical isolates of RGM strains *M. abscessus*, *M. fortuitum*, and *M. chelonae* were collected from the Medical Laboratory Department of the E-Da Hospital. *M. smegmatis* ATCC 14468 was a reference strain of RGM used in the antibacterial susceptibility testing. Mycobacteria were cultured in Middlebrook 7H9 Broth (BD Biosciences, NJ, USA) supplemented with 10% oleic albumin dextrose catalase (OADC) enrichment (Creative Life Science CO., LTD., New Taipei City, Taiwan) and 0.5% glycerol.

### Antibacterial susceptibility testing and determination of MIC)

Antibacterial susceptibility testing of 11 antibiotics against RGM clinical strains was determined using broth microdilution-based automated AST system with SENSITITRE RAPMYCOI panel (Trek Diagnostics/Thermo Fisher, Bremen, Germany) according to the instructions of the manufacturer, and the susceptible and resistant breakpoints used were those recommended by the Clinical and Laboratory Standards Institute (CLSI) guidelines (45). MIC values of naturally occurring compounds were measured using cation-adjusted Mueller-Hinton broth (CAMHB; BD Biosciences, NJ, USA) with a broth microdilution method. The ranges of tested concentrations were 16–2,048 μg/mL for *trans*-cinnamaldehyde, carvacrol, citral, and geraniol, and 8–1,024 μg/mL for gentisaldehyde and phloroglucinaldehyde. The MIC was defined as the minimum concentration of the test agent at which there was no visible growth of the test strain.

### Disk diffusion assay

The Kirby-Bauer disc diffusion method was used to determine inhibition zone size caused by compounds against RGM strains (46). Each disc (diameter 8 mm) contained 20 µL of test compounds that were placed on Muller-Hinton Agar (MHA) (BD Biosciences, NJ, USA) plates inoculated with 5 × 10^5^ cfu/mL of bacteria. The zone of inhibition was determined after incubation at 37°C for 5 days. Liquid compounds were tested for the highest concentrations: *trans*-cinnamaldehyde 99 mg/mL, carvacrol 39.2 mg/mL, citral 95 mg/mL, and geraniol 98 mg/mL. The tested concentrations for solid compounds were stock solutions at 50 mg/mL (gentisaldehyde, phloroglucinaldehyde, capsaicin, caffeic acid, chlorogenic acid, vanillic acid, berberine chloride, and palmatine chloride). Reference antibiotic amikacin (3 mg/mL) was tested for comparison, of which the tested quantity in the discs was about 1/13–1/33 of the compound quantity. In this study, disc diffusion assays were used to identify potential inhibitory agents. The antimicrobial activities of anti-RGM compounds were further assessed and compared based on MIC determination.

### Time-kill assay

The time-kill kinetics of antimicrobial agents against RGM were performed based on the method described (47). Briefly, individual tubes of 20 mL of CAMHB plus OADC containing test compounds or antimicrobial agents (at the concentrations as indicated) were inoculated with 100 µL RGM bacterium suspension to the final concentration of ~10^6^ cfu/mL and incubated at 37°C under shaking condition (100 rpm). A growth control tube, with inoculum but without antimicrobial agents, was included. At the defined time intervals (0, 24, 48, 72, 96, or 120 h), bacterial numbers were quantified by plating dilutions on 7H11 agar (BD Biosciences, NJ, USA) and counting colony-forming units (CFUs). Bactericidal activity is defined as greater than 3 log_10_-fold decrease in CFUs/mL (surviving bacteria), which is equivalent to 99.9% killing of the initial inoculum (48). Synergy of an antimicrobial combination was defined as a ≧2 log_10_ decrease in CFUs/mL compared with the most active single agent at any time point (26, 49).

### Biofilm assay

Biofilm formation of mycobacteria was quantified according to the methods described previously (50). Briefly, RGM suspensions at a final concentration of 10^7^ CFU/mL were prepared in 0.9% sodium chloride from fresh cultures in 7H11 agar and 10-fold diluted in 7H9 broth. Two hundred microliters was distributed to each well of 96-well polypropylene plates for incubation at 37°C to allow biofilm formation for 5 days. The contents of each well were removed and each well was vigorously washed three times with sterile distilled water, followed by staining with 200 µL crystal violet (1%) at room temperature for 15 min. Each well was then washed with distilled water three times to remove excess dye and allowed to dry at room temperature. The crystal violet was dissolved in 200 µL of 95% ethanol and the optical density at 570 nm was measured.

### Checkerboard assay

The synergistic interaction of compounds in combination with antibiotics was analyzed using the checkerboard assay, which was commonly used to assess antimicrobial combinations *in vitro* (49). Serial dilutions of naturally occurring compounds and antibiotics were mixed in each well of a 96-well microplate. Fifty-microliter aliquots of the first and second antimicrobial agents were added in vertical and horizontal orientation, respectively. A 100 µL of fresh bacterial suspension (1 × 10^6^ cfu/mL) was added to each well and incubated for 5 days. Fractional inhibitory concentration indexes (FICIs) were calculated: FICI = (MIC of antimicrobial agent A in combination/MIC of antimicrobial agent A alone) + (MIC of antimicrobial agent B in combination/MIC of antimicrobial agent B alone). The FICI index was interpreted as synergistic when it was ≦0.5, additive when it was >0.5–1, indifferent when it was >1–4.0, and antagonistic when it was >4 (49).

## SUPPLEMENTAL MATERIAL

Supplemental material spectrum.00199-24-s0001.pdf Table S1; Fig. S1 and S2.

ASM does not own the copyrights to Supplemental Material that may be linked to, or accessed through, an article. The authors have granted ASM a non-exclusive, world-wide license to publish the Supplemental Material files. Please contact the corresponding author directly for reuse.

## References

[1] Griffith DE, Aksamit T, Brown-Elliott BA, Catanzaro A, Daley C, Gordin F, Holland SM, Horsburgh R, Huitt G, Iademarco MF, Iseman M, Olivier K, Ruoss S, von Reyn CF, Wallace RJ, Winthrop K, ATS Mycobacterial Diseases Subcommittee, American Thoracic Society, Infectious Disease Society of America. 2007. An official ATS/IDSA statement: diagnosis, treatment, and prevention of nontuberculous mycobacterial diseases. Am J Respir Crit Care Med 175:367–416. doi:10.1164/rccm.200604-571ST 17277290

[2] Brown-Elliott BA, Philley JV. 2017. Rapidly growing mycobacteria. Microbiol Spectr 5. doi:10.1128/microbiolspec.TNMI7-0027-2016 PMC11687460 28084211

[3] Mertaniasih NM, Kusumaningrum D, Koendhori EB, Kusmiati T, Dewi DNSS, Soedarsono. 2017. Nontuberculous mycobacterial species and Mycobacterium tuberculosis complex coinfection in patients with pulmonary tuberculosis in Dr. Soetomo Hospital, Surabaya, Indonesia. Int J Mycobacteriol 6:9–13. doi:10.4103/2212-5531.201894 28317798

[4] Winthrop KL, McNelley E, Kendall B, Marshall-Olson A, Morris C, Cassidy M, Saulson A, Hedberg K. 2010. Pulmonary nontuberculous mycobacterial disease prevalence and clinical features: an emerging public health disease. Am J Respir Crit Care Med 182:977–982. doi:10.1164/rccm.201003-0503OC 20508209

[5] Griffith DE, Stout JE. 2010. It is better to light a candle... than to repeat the opinions of experts. Am J Respir Crit Care Med 182:865–866. doi:10.1164/rccm.201008-1251ED 20884939

[6] Tu H-Z, Lee H-S, Chen Y-S, Lee S-J. 2022. High rates of antimicrobial resistance in rapidly growing mycobacterial infections in Taiwan. Pathogens 11:969. doi:10.3390/pathogens11090969 36145400 PMC9504488

[7] Kumar C, Shrivastava K, Singh A, Chauhan V, Varma-Basil M. 2021. Skin and soft-tissue infections due to rapidly growing mycobacteria: an overview. Int J Mycobacteriol 10:293–300. doi:10.4103/ijmy.ijmy_110_21 34494569

[8] Johansen MD, Herrmann JL, Kremer L. 2020. Non-tuberculous mycobacteria and the rise of Mycobacterium abscessus. Nat Rev Microbiol 18:392–407. doi:10.1038/s41579-020-0331-1 32086501

[9] van Ingen J, Boeree MJ, Dekhuijzen PNR, van Soolingen D. 2009. Environmental sources of rapid growing nontuberculous mycobacteria causing disease in humans. Clin Microbiol Infect 15:888–893. doi:10.1111/j.1469-0691.2009.03013.x 19845700

[10] Lai CC, Tan CK, Chou CH, Hsu HL, Liao CH, Huang YT, Yang PC, Luh KT, Hsueh PR. 2010. Increasing incidence of nontuberculous mycobacteria, Taiwan, 2000-2008. Emerg Infect Dis 16:294–296. doi:10.3201/eid1602.090675 20113563 PMC2958002

[11] Shrivastava K, Kumar C, Singh A, Narang A, Giri A, Sharma NK, Gupta S, Chauhan V, Gunasekaran J, Balasubramanian V, Chaudhry A, Singla R, Prasad R, Varma-Basil M. 2020. An overview of pulmonary infections due to rapidly growing mycobacteria in South Asia and impressions from a subtropical region. Int J Mycobacteriol 9:62–70. doi:10.4103/ijmy.ijmy_179_19 32474491

[12] Hatakeyama S, Ohama Y, Okazaki M, Nukui Y, Moriya K. 2017. Antimicrobial susceptibility testing of rapidly growing mycobacteria isolated in Japan. BMC Infect Dis 17:197. doi:10.1186/s12879-017-2298-8 28270102 PMC5341166

[13] Yang SC, Hsueh PR, Lai HC, Teng LJ, Huang LM, Chen JM, Wang SK, Shie DC, Ho SW, Luh KT. 2003. High prevalence of antimicrobial resistance in rapidly growing mycobacteria in Taiwan. Antimicrob Agents Chemother 47:1958–1962. doi:10.1128/AAC.47.6.1958-1962.2003 12760874 PMC155839

[14] Huang T-S, Lee SS-J, Hsueh P-R, Tsai H-C, Chen Y-S, Wann S-R, Leu H-S, Ko W-C, Yan J-J, Yuan S-Z, Chang F-Y, Lu J-J, Wang J-H, Wang H-K, Liu Y-C. 2008. Antimicrobial resistance of rapidly growing mycobacteria in western Taiwan: SMART program 2002. J Formos Med Assoc 107:281–287. doi:10.1016/s0929-6646(08)60088-1 18445541

[15] Jarand J, Levin A, Zhang L, Huitt G, Mitchell JD, Daley CL. 2011. Clinical and microbiologic outcomes in patients receiving treatment for Mycobacterium abscessus pulmonary disease. Clin Infect Dis 52:565–571. doi:10.1093/cid/ciq237 21292659

[16] Martín-de-Hijas NZ, García-Almeida D, Ayala G, Fernández-Roblas R, Gadea I, Celdrán A, Gómez-Barrena E, Esteban J. 2009. Biofilm development by clinical strains of non-pigmented rapidly growing mycobacteria. Clin Microbiol Infect 15:931–936. doi:10.1111/j.1469-0691.2009.02882.x 19624503

[17] Falkinham JO. 2009. Surrounded by mycobacteria: nontuberculous mycobacteria in the human environment. J Appl Microbiol 107:356–367. doi:10.1111/j.1365-2672.2009.04161.x 19228258

[18] Vaerewijck MJM, Huys G, Palomino JC, Swings J, Portaels F. 2005. Mycobacteria in drinking water distribution systems: ecology and significance for human health. FEMS Microbiol Rev 29:911–934. doi:10.1016/j.femsre.2005.02.001 16219512

[19] Friedman M, Henika PR, Levin CE, Mandrell RE. 2004. Antibacterial activities of plant essential oils and their components against Escherichia coli O157:H7 and Salmonella enterica in apple juice. J Agric Food Chem 52:6042–6048. doi:10.1021/jf0495340 15366861

[20] Friedman M, Henika PR, Mandrell RE. 2003. Antibacterial activities of phenolic benzaldehydes and benzoic acids against Campylobacter jejuni, Escherichia coli, Listeria monocytogenes, and Salmonella enterica. J Food Prot 66:1811–1821. doi:10.4315/0362-028x-66.10.1811 14572218

[21] Newton SM, Lau C, Wright CW. 2000. A review of antimycobacterial natural products. Phytother Res 14:303–322. doi:10.1002/1099-1573(200008)14:5<303::aid-ptr712>3.0.co;2-n 10925394

[22] Sadeer NB, Mahomoodally MF. 2021. Antibiotic potentiation of natural products: a promising target to fight pathogenic bacteria. Curr Drug Targets 22:555–572. doi:10.2174/1389450121666200924113740 32972338

[23] Vaou N, Stavropoulou E, Voidarou CC, Tsakris Z, Rozos G, Tsigalou C, Bezirtzoglou E. 2022. Interactions between medical plant-derived bioactive compounds: focus on antimicrobial combination effects. Antibiotics (Basel) 11:1014. doi:10.3390/antibiotics11081014 36009883 PMC9404952

[24] Evaluations of the Joint FAO/WHO Expert Committee on Food Additives (JECFA). Report TRS 901-JECFA 55/22, TRS 901-JECFA 55/44, TRS 922-JECFA 61/75, TRS 922-JECFA 61/75. https://apps.who.int/food-additives-contaminants-jecfa-database/.

[25] Schabauer A, Zutz C, Lung B, Wagner M, Rychli K. 2018. Gentisaldehyde and its derivative 2,3-dihydroxybenzaldehyde show antimicrobial activities against bovine mastitis Staphylococcus aureus. Front Vet Sci 5:148. doi:10.3389/fvets.2018.00148 30050910 PMC6050399

[26] Gómara-Lomero M, López-Calleja AI, Rezusta A, Aínsa JA, Ramón-García S. 2023. In vitro synergy screens of FDA-approved drugs reveal novel zidovudine- and azithromycin-based combinations with last-line antibiotics against Klebsiella pneumoniae. Sci Rep 13:14429. doi:10.1038/s41598-023-39647-9 37660210 PMC10475115

[27] Wong SYY, Grant IR, Friedman M, Elliott CT, Situ C. 2008. Antibacterial activities of naturally occurring compounds against Mycobacterium avium subsp. paratuberculosis. Appl Environ Microbiol 74:5986–5990. doi:10.1128/AEM.00981-08 18676709 PMC2565950

[28] Vasconcelos NG, Croda J, Simionatto S. 2018. Antibacterial mechanisms of cinnamon and its constituents: a review. Microb Pathog 120:198–203. doi:10.1016/j.micpath.2018.04.036 29702210

[29] Doyle AA, Stephens JC. 2019. A review of cinnamaldehyde and its derivatives as antibacterial agents. Fitoterapia 139:104405. doi:10.1016/j.fitote.2019.104405 31707126

[30] Sawicki R, Golus J, Przekora A, Ludwiczuk A, Sieniawska E, Ginalska G. 2018. Antimycobacterial activity of cinnamaldehyde in a Mycobacterium tuberculosis(H37Ra). Molecules 23:2381. doi:10.3390/molecules23092381 30231479 PMC6225461

[31] Mączka W, Twardawska M, Grabarczyk M, Wińska K. 2023. Carvacrol-A natural phenolic compound with antimicrobial properties. Antibiotics (Basel) 12:824. doi:10.3390/antibiotics12050824 37237727 PMC10215463

[32] Hyldgaard M, Mygind T, Meyer RL. 2012. Essential oils in food preservation: mode of action, synergies, and interactions with food matrix components. Front Microbiol 3:12. doi:10.3389/fmicb.2012.00012 22291693 PMC3265747

[33] Nakamura de Vasconcelos SS, Caleffi-Ferracioli KR, Hegeto LA, Baldin VP, Nakamura CV, Stefanello TF, Freitas Gauze G de, Yamazaki DA, Scodro RB, Siqueira VL, Cardoso RF. 2018. Carvacrol activity & morphological changes in Mycobacterium tuberculosis. Future Microbiol 13:877–888. doi:10.2217/fmb-2017-0232 29877104

[34] He TF, Wang LH, Niu DB, Wen QH, Zeng XA. 2019. Cinnamaldehyde inhibit Escherichia coli associated with membrane disruption and oxidative damage. Arch Microbiol 201:451–458. doi:10.1007/s00203-018-1572-5 30293114

[35] Wendakoon CN, Sakaguchi M. 1995. Inhibition of amino acid decarboxylase activity of Enterobacter aerogenes by active components in spices. J Food Prot 58:280–283. doi:10.4315/0362-028X-58.3.280 31137282

[36] Gill AO, Holley RA. 2006. Inhibition of membrane bound ATPases of Escherichia coli and Listeria monocytogenes by plant oil aromatics. Int J Food Microbiol 111:170–174. doi:10.1016/j.ijfoodmicro.2006.04.046 16828188

[37] Nazzaro F, Fratianni F, De Martino L, Coppola R, De Feo V. 2013. Effect of essential oils on pathogenic bacteria. Pharmaceuticals (Basel) 6:1451–1474. doi:10.3390/ph6121451 24287491 PMC3873673

[38] Nostro A, Filocamo A, Giovannini A, Catania S, Costa C, Marino A, Bisignano G. 2012. Antimicrobial activity and phenolic content of natural site and micropropagated Limonium avei (De Not.) Brullo & Erben plant extracts. Nat Prod Res 26:2132–2136. doi:10.1080/14786419.2011.628669 22014177

[39] Nostro A, Papalia T. 2012. Antimicrobial activity of carvacrol: current progress and future prospectives. Recent Pat Antiinfect Drug Discov 7:28–35. doi:10.2174/157489112799829684 22044355

[40] Marini E, Di Giulio M, Ginestra G, Magi G, Di Lodovico S, Marino A, Facinelli B, Cellini L, Nostro A. 2019. Efficacy of carvacrol against resistant rapidly growing mycobacteria in the planktonic and biofilm growth mode. PLoS One 14:e0219038. doi:10.1371/journal.pone.0219038 31260476 PMC6602199

[41] Langeveld WT, Veldhuizen EJA, Burt SA. 2014. Synergy between essential oil components and antibiotics: a review. Crit Rev Microbiol 40:76–94. doi:10.3109/1040841X.2013.763219 23445470

[42] Endo A, Hanawa K, Nemoto A, Ishikawa T, Kazama S, Kagami Y, Maebayashi Y, Katsumata N, Naito A, Kobayashi Y, Kawano Y, Hanawa T. 2022. Evaluation of nephrotoxicity and ototoxicity following amikacin administration once daily or every 48 hours in neonates. Medicine 101:e31425. doi:10.1097/MD.0000000000031425 36316882 PMC9622663

[43] LiverTox: clinical and research information on drug-induced liver injury. 2012. Bethesda (MD) National Institute of Diabetes and Digestive and Kidney Diseases 31643176

[44] Drugs and lactation database (LactMed). 2022. Clarithromycin. Bethesda (MD) National Institute of Child Health and Human Development

[45] Clinical and Laboratory Standards Institute Institute. 2011. CLSI Document M24-A2. Susceptibility testing of mycobacteria, nocardiae, and other aerobic actinomycetes. Approved standard. 2nd ed. Clinical and Laboratory Standards Institute, Wayne, PA.

[46] Singh R, Hussain S, Verma R, Sharma P. 2013. Anti-mycobacterial screening of five Indian medicinal plants and partial purification of active extracts of Cassia sophera and Urtica dioica. Asian Pac J Trop Med 6:366–371. doi:10.1016/S1995-7645(13)60040-1 23608375

[47] Ferro BE, van Ingen J, Wattenberg M, van Soolingen D, Mouton JW. 2015. Time-kill kinetics of antibiotics active against rapidly growing mycobacteria. J Antimicrob Chemother 70:811–817. doi:10.1093/jac/dku431 25344808

[48] Clinical and Laboratory Standards Institute Institute. 1999. CLSI document M26-A. Clinical and Laboratory Standards Institute, Wayne, PA.

[49] Al-Ani I, Zimmermann S, Reichling J, Wink M. 2015. Pharmacological synergism of bee venom and melittin with antibiotics and plant secondary metabolites against multi-drug resistant microbial pathogens. Phytomedicine 22:245–255. doi:10.1016/j.phymed.2014.11.019 25765829

[50] Sousa S, Bandeira M, Carvalho PA, Duarte A, Jordao L. 2015. Nontuberculous mycobacteria pathogenesis and biofilm assembly. Int J Mycobacteriol 4:36–43. doi:10.1016/j.ijmyco.2014.11.065 26655196

