# Supplementary material for: Anti-biofilm activities and antibiotic synergy of naturally occurring compounds against drug-resistant rapidly growing mycobacteria
Source: Microbiol Spectr. 2024 Jun 27;12(8):e00199-24. doi: 10.1128/spectrum.00199-24 (PMC11302017; doi:10.1128/spectrum.00199-24)
Supplement: Supplemental material — Table S1; Fig. S1 and S2. [file spectrum.00199-24-s0001.pdf]

Supplementary

Table S1. LD_{50} (lethal dose, 50%) values for *in vivo* acute toxicity of anti-RGM compounds.

**Compound**	**Test organism**	**Route**	**LD_{50} (mg/kg)**	**References**
Cinnamaldehyde	Rat	Oral	2220	Food and Cosmetics Toxicology, 1964
Carvacrol	Rat	Oral	810	Food and Cosmetics Toxicology, 1964
Citral	Rat	Oral	4960	Food and Cosmetics Toxicology, 1964
Geraniol	Rat	Oral	3600	Food and Cosmetics Toxicology, 1964
Phloroglucinaldehyde	Mouse	Oral	3200	Biochemical Journal, 1940

Source: PubChem (https://pubchem.ncbi.nlm.nih.gov/).

Supplementary figures

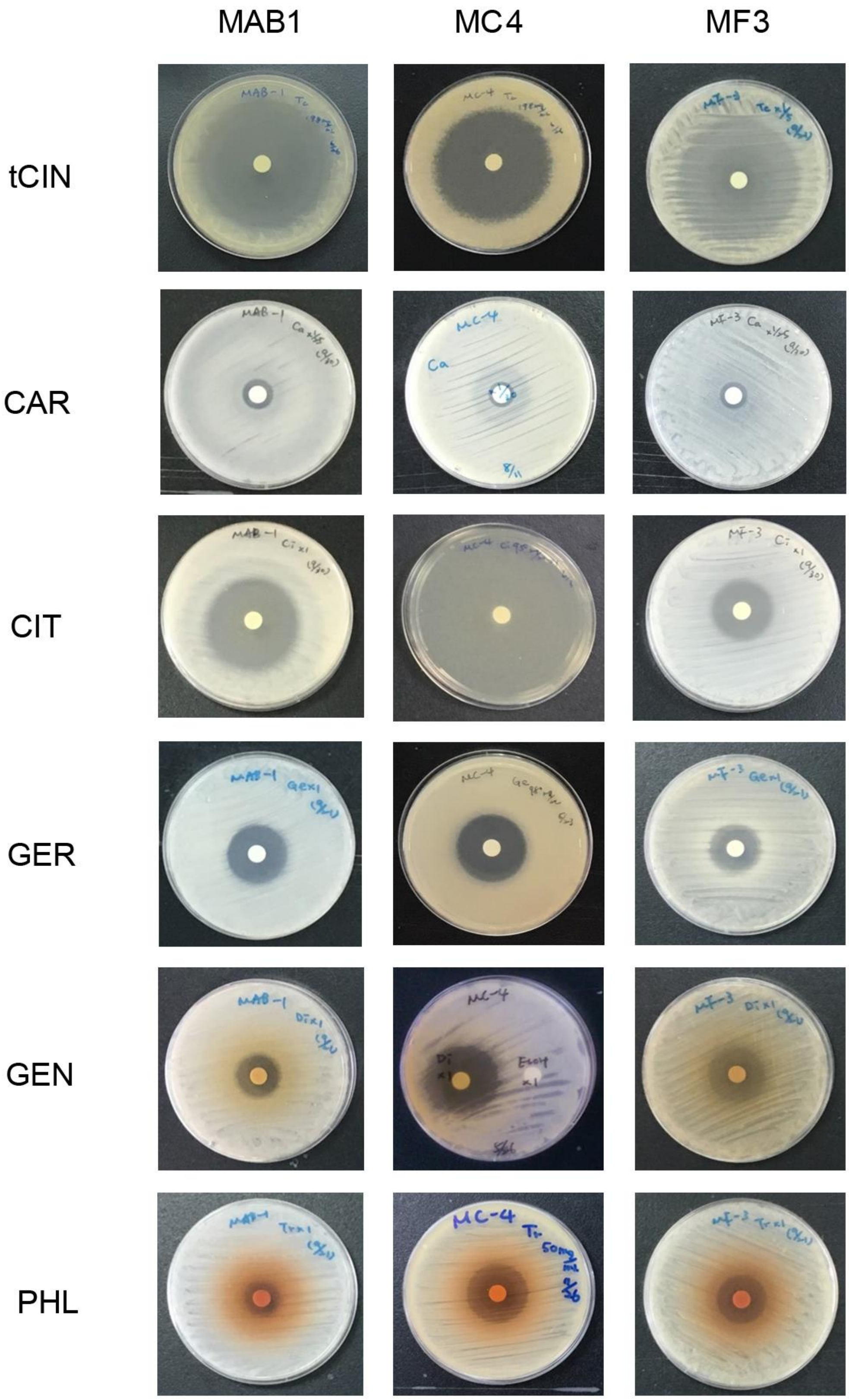

Figure S1. Representative graphs showing inhibition zones on clinical RGM by naturally occurring compounds in a disk diffusion assay: (A) *M. abscessus* MAB1; (B) *M. chelonae* MC4; (C) *M. fortuitum* MF3. Tested compounds: tCIN, *trans*-cinnamaldehyde (99 mg/ml); CAR, carvacrol (39.2 mg/ml); CIT, citral (95 mg/ml); GER, geraniol (98 mg/ml); GEN, gentisaldehyde (50 mg/ml); PHL, phloroglucinaldehyde (50 mg/ml). Paper disk diameter 8 mm; test volume 20 μL.

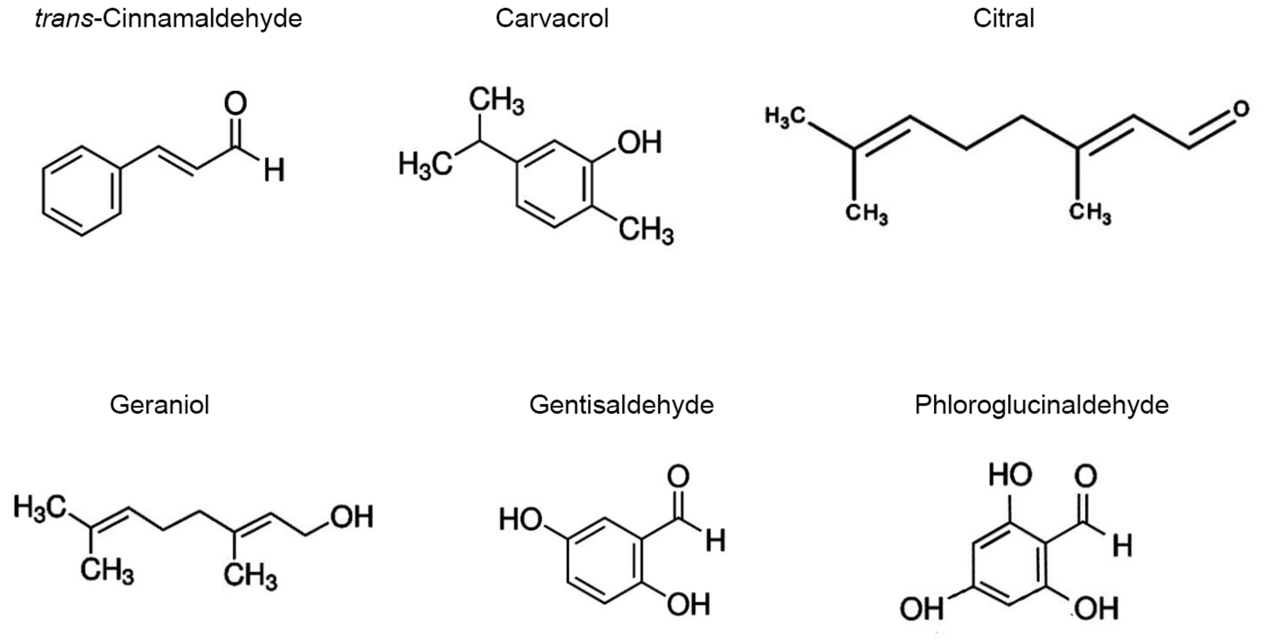

Figure S2. Structures of identified anti-RGM compounds.
